# Evolution of Habitat Quality and Its Response to Topographic Gradient Effect in a Karst Plateau: A Case Study of the Key Biodiversity Conservation Project Area of Wuling Mountains

**DOI:** 10.3390/ijerph20010331

**Published:** 2022-12-26

**Authors:** Bo Xie, Shunbing Meng, Mingming Zhang

**Affiliations:** 1 College of Forestry, Guizhou University, Guiyang 550025, China; 2 Research Center for Biodiversity and Nature Conservation of Guizhou University, Guiyang 550025, China

**Keywords:** habitat quality, InVEST model, landscape pattern, land use change, topographic gradient

## Abstract

Habitat quality (HQ) is widely considered to be a proxy indicator for biodiversity. Assessing the dynamic changes in HQ induced by land-use and land-cover (LULC) changes could provide a scientific perspective for regional sustainable development, especially in the ecologically fragile karst plateau mountainous areas. We selected nine landscape metrics to examine LULC dynamics in the Key Biodiversity Conservation Project Area of Wuling Mountains in Guizhou province, China, based on the land-use data for the 1990–2018 period. HQ was evaluated using the InVEST model and the topographic gradient effect on HQ was analyzed using the topographic position index. The results showed that the dominant land categories in the study area were arable land, grassland, and forestland. Land transformation was mainly characterized by a shift from cultivated land to construction land and forestland, and a mutual conversion between grassland and forestland. The changes improved landscape heterogeneity and the degree of fragmentation. The HQ of the study area was high, although exhibited a declining trend from 1990 to 2018. The eastern and western parts had relatively high HQ, whereas the southern and northern parts had low HQ. The topographic gradient had a significant effect on spatial patterns of HQ. High HQ distribution was consistent with that of forestland and was dominant along the topographic gradient, while low HQ distribution was consistent with that of construction land and cultivated land and was dominant along the topographic gradient.

## 1. Introduction

Habitat quality (HQ) refers to the ability of ecosystems to supply and support essential goods and services for both individuals and groups [1,2], which is reflected in the status of regional biodiversity to a certain extent [3,4]. Biodiversity includes the diversity of animals, plants, and microorganisms at the genetic, species, and ecosystem levels, providing regulating, supporting, and cultural ecosystem services [5]. The increase in human activities has led to widespread biodiversity loss and disintegration of ecological and socioeconomic systems [6,7], which harm the well-being of local communities [8]. In developing countries, the pressure is even greater, with the majority of the poor being dependent on natural resources for their livelihoods [9]. Land-use and land-cover (LULC) changes are essential manifestations of human activities, which pose a significant risk factor for HQ [10,11]. LULC changes, including changes in proportions, structures, and intensity, fundamentally alter the composition and configuration of ecosystems and, ultimately, influence the energy flow and material circulation between habitat patches [12,13]. Therefore, habitat conservation is increasingly threatened by anthropogenic impacts, particularly human-dominated LULC changes that are regarded as a key factor influencing HQ decline [14].

HQ is a significant foundation for ecosystem service capacity and biodiversity maintenance [15]. Early research on habitats mainly focused on the habitat status of specific species and habitat impacts on species [16,17]. With the development of environmental science, many models, such as the HIS model [18], the Integrated Valuation of Ecosystem Services and Trade-offs (InVEST) model [2], and the Social Values for Ecosystem Services (SolVES) model [19], have emerged to efficiently calculate HQ attributes at various scales. The “HQ” module in the InVEST model (InVEST-HQ) is a widely used ecological function assessment model [20] that assesses HQ by combining habitat suitability with anthropogenic threats to biodiversity and provides more detailed information regarding biodiversity status [17,21]. The main advantages of the InVEST-HQ are that the assessment module parameters are readily available and the results are accurately analyzed, thereby reflecting habitat distribution and degradation status of different landscape types [22].

Topography is an important natural factor, which substantially influences the spatial pattern differentiation of ecosystem services and HQ [23]. Furthermore, terrain factors have major impacts on nutrient exchange and energy circulation in habitat systems [24]. Numerous studies have focused on the impact of land-use change on HQ in watersheds [18], hilly areas [22], and cities [25] to highlight the relationship between HQ and land use, and landscape patterns and topographic factors [26,27]. However, few studies have explored variations in HQ with topographic gradients.

The Key Biodiversity Conservation Project Area of Wuling Mountains (KBCPW) is an important region for ecological protection and restoration located in Guizhou province, China. The KBCPW is a typical karst plateau mountain area characterized by a unique landform consisting of mountains and hills. As an important part of the terrestrial ecosystem, mountainous areas are rich in biodiversity and provide strong support for regional economic development. However, plateau ecosystems are exposed to many risks, such as habitat loss and environmental degradation [28]. The ecological environment in mountainous areas is increasingly becoming vulnerable to the irrational use of resources by humans, which has led to several ecological problems and hindered local economic development. The distribution of mountainous habitats is influenced more by topographic factors than that of plain agricultural areas. Therefore, an assessment of HQ in ecologically sensitive mountainous areas from a topographic perspective is crucial for understanding the spatiotemporal differentiation characteristics of HQ and enhancing effective ecosystem management.

The protection and maintenance of biodiversity, as well as satisfaction of human demands, have become of increasing concern [29]. Understanding the response of HQ to LULC change is important for maintaining biodiversity and land management; therefore, it is necessary for policymakers to predict habitat protection based on the exploration of habitats changes [30]. In this study, we mapped and identified the HQ of the KBCPW and analyzed the variations in HQ during the 1900–2018 period based on the land-use data from 1990, 2000, 2010, and 2018. Subsequently, the terrain gradient effect on HQ was analyzed to reveal the variations in HQ using the topographic position index.

## 2. Data and Methods

### 2.1. Study Area

**Figure 1:**
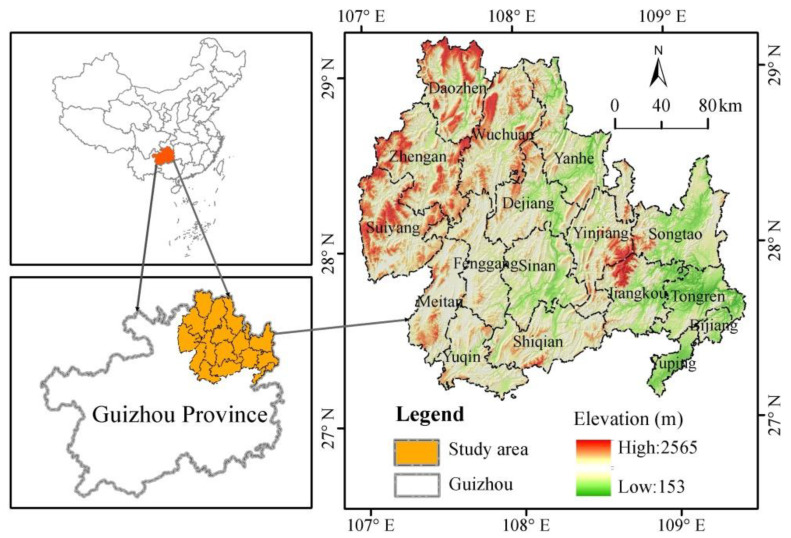
Geographical location of the KBCPW.

The KBCPW, which is located in the northeastern part of Guizhou Province, is an important area for biodiversity conservation and sustainable economic and social development. The KBCPW covers 17 counties (cities and districts), with an area of 33451 km^2^, which accounts for 18.99% of the total land area in Guizhou Province. The region aims to support the integrated protection and restoration of landscapes, forests, fields, lakes, and grasslands. The Fanjing Mountain and its surrounding areas are core areas of the KBCPW. The altitude ranges from 153 to 2565 m, and the terrain is high in the northwestern and low in the southeastern regions (Figure 1), with the highest peak being located in the eastern part of the Fanjing Mountain. The KBCPW is located in a low-latitude zone and belongs to the subtropical humid monsoon climate. The area receives abundant annual precipitation of 900–1300 mm that is mainly concentrated in spring and summer, whereas the annual average temperature is 14–16 °C.

Guizhou is a unique mountainous province in China without plains and its ecological environment is fragile, experiencing widespread ecological and environmental problems, as well as heavy soil erosion and karst rocky desertification. In particular, in recent decades, the increasing intensity of human activities, such as urban expansion and rural development, has led to changes in LULC, which have exerted considerable pressure on the ecosystem, resulting in changes in the structure of the regional landscapes and increase in the complexity of landscape attributes, and consequently, negatively impacting the habitats and sustainable development of the area.

### 2.2. Data Resources

The land-use data used in this study covered the years 1990, 2000, 2010, and 2018 and were obtained from the Data Center for Resources and Environmental Sciences of the Chinese Academy of Sciences, with a spatial resolution of 30 m × 30 m (http://www.resdc.cn). The land-use types were classified into six first-class types (i.e., arable land, forestland, grassland, water, construction land, and unused land), and 25 second-class types, although only 16 of the 25 second-class land types were observed in the KBCPW (Table 1). The comprehensive evaluation accuracy of the data was more than 90%, which satisfied the requirements of the study. Digital elevation model (DEM) data were acquired using the ASTER Global Digital Elevation Map (v 2) from the Geospatial Data Cloud with a resolution of 30 m (http://www.gscloud.cn/).

### 2.3. Methods

#### 2.3.1. Selection of Landscape Pattern Index

Analysis of landscape change patterns is vital for studying ecological processes and ecosystem function changes in mountain landscapes. Landscape patch size, connectivity, and landscape diversity have important implications for biodiversity and various ecological processes [22]. Landscape metrics are quantitative indicators comprising centralized landscape pattern information [31]. Specifically, landscape patterns are classified at the landscape and class levels. Although the ecological significance of landscape indicators varies, strong correlations are often observed between some indicators. In the present study, we selected 10 landscape metrics to assess the LULC classification patterns as follows: number of patches (NP), patch density (PD), mean patch area (AREA_MN), largest patch index (LPI), landscape shape index (LSI), contagion index (CONTAG), COHESION index (COHESION), aggregation index (AI), Shannon’s diversity index (SHDI), and Shannon’s evenness index (SHEI). These landscape pattern indices were calculated using FRAGSTATS (v 4.2) [32]. Details of the specific indices and calculations are presented in the methodology of the original articles [33,34].

#### 2.3.2. The InVEST-HQ Model

The InVEST model (v 3.9.0) was used to assess the spatiotemporal changes in HQ. Based on the impact of human activities on LULC, HQ maps are generated by combining habitat suitability of land-use type and factors threatening biodiversity. The module is based on the assumption that LULC with a high HQ value is capable of supporting more biodiversity, whereas a low HQ value indicates biodiversity loss. HQ is reflected by the HQ index, which is calculated as follows [35]:

(1) $$Q_{xj}=H_{j}\left[1-\left(\frac{D_{xj}^{z}}{D_{xj}^{z}+K^{z}}\right)\right]\;$$

where *Q_xj_* is the HQ of raster *x* in land-use type *j*; *D_xj_* is the threat level of raster x in land-use type *j*; H_j_ is the habitat suitability of land-use type *j*; and *k* is half the saturation constant (which is half of the maximum value of *D_xj_*).

(2) $$D_{xj}=\overset{R}{\underset{r=1}{\sum }}\overset{Y_{r}}{\underset{y=1}{\sum }}\left(\omega_{r}/\overset{R}{\underset{r=1}{\sum }}\omega_{r}\right)r_{y}i_{rxxy}\beta_{x}S_{jr}$$

where *R* is the number of stress factors; *Y_r_* is the total number of grid cells of stress factors; *ω_r_* is the weight; *r_y_* is the number of stress factors on the grid cells; *β_x_* is the accessibility level of grid *x*; *S_jr_* is the sensitivity of land-use type *j* to stress factors, with a value range of 0–1; and *i_rxy_* is the influence distance of stress factors.

(3) $$i_{rxy}=1-\left(d_{xy}/d_{rmax}\right)$$

(4) $$i_{rxy}=1-\left(d_{xy}/d_{rmax}\right)$$

where *d_xy_* is the distance between grid *x* and grid *y*, and *d_r_*_max_ is the influencing scope of the threat factor *r*.

The data inputs (spatial and nonspatial) required to run the InVEST-HQ model include the LULC maps from multiple dates, threat sources and impacts, habitat types, habitat sensitivity to the threats, and the half-saturation constant. In this study, cultivated and construction lands were selected as the habitat threat factors because of the greater interference caused by human activities on cultivated and construction lands. Second, the study area is a typical karst landform, mainly characterized by mountainous and hilly plateaus and scattered distribution of rural settlements; therefore, construction land was converted to urban construction land, rural settlements, and other construction land. The relative weight of the threat factors, the maximum distance between habitats and each threat source, and the habitat sensitivity of each threat were determined based on the model manual [35] and literature review [36,37] (Table 2 and Table 3).

#### 2.3.3. Terrain and Distribution Indices

The topography of the study area is complex, considering that topographical conditions have a greater impact on land use, landscape patterns, and HQ [38,39]. Therefore, the present study used the topographic position index to determine the topographic gradient, according to the following equation:

(5) $$T=\log\left[\left(E/E+1\right)\times \left(S/S+1\right)\right]$$

where T represents the topographic position index; E and $\bar{E}$ are the elevation and average elevation values (m) of the study area, respectively; and S and $\bar{S}$ are the slope and average slope values (◦) of the study area, respectively. In general, the topographic position index is high when the elevation and slope values are high and low when the elevation and slope values are low. The topographic position index is moderate when the elevation value is high but the slope value is low or when the elevation value is low but the slope value is high. The terrain index was calculated using the DEM data, and then we divided the index into five levels using ArcGIS according to the equal spacing classification method.

The distribution index [40] can reflect the influence of topographic conditions on the spatial distribution of landscape components; that is, it reflects the frequency of occurrence of different landscape components along the topographic gradient. To further analyze the topographic gradient effect of HQ in the KBCPW, the topographic distribution index was used to investigate the various characteristics of HQ with terrain factors, according to the following equation [41]:

(6) $$P=\left(A_{\mathrm{ie}}/A_{i}\right)/\left(A_{e}/A\right)$$

where P represents the distribution index; A_ie_ is the area of Grade Ⅰ habitat mass within the terrain gradient of Grade e; A_i_ is the total area of the Grade Ⅰ HQ; A_e_ is the total area within the e-level terrain gradient; and A is the total area of the study area. A higher P value indicates a more pronounced advantage of the quality of a certain stage within the terrain-level gradient of that stage. A P value > 1 indicates a dominant position, whereas a P value < 1 indicates a disadvantage [39].

## 3. Results

### 3.1. Land-Use Change Analysis

**Figure 2:**
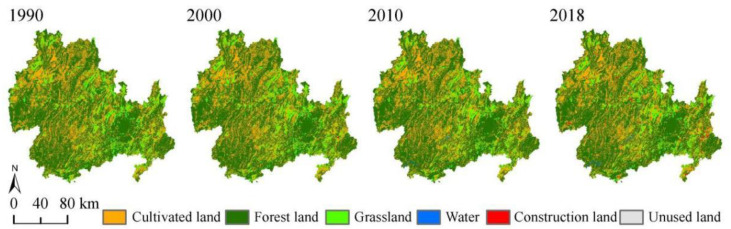
Land-use change in the KBCPW from 1990 to 2018.

The LULC in the KBCPW in Guizhou Province exhibited varying trends between 1990 and 2018 (Figure 2). The main types of land use were arable land, grassland, and forestland, accounting for 90%. In addition, forestland and grassland decreased by 134.4 km^2^ and 241 km^2^, respectively. Conversely, construction land increased significantly, with a substantial increase of 4.5-fold (from 0.18% in 1990 to 0.82% in 2018) being observed in the proportion of land area in 2018 when compared to that in 1990. Cultivated land and water area increased slightly by 77 km^2^ and 84.7 km^2^, respectively. However, the area of unused land exhibited insignificant changes (Table 4).

**Figure 3:**
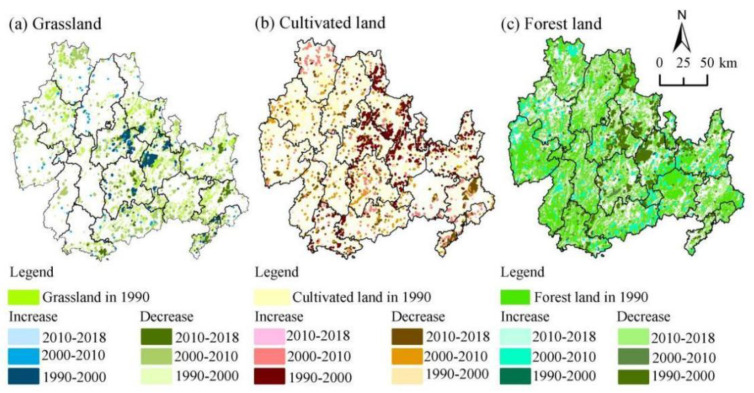
Spatial patterns of changes in major land-use types during the 1990–2018 period.

The transfer matrix of each LULC was constructed for the four periods from 1990 to 2018 (Table 5). During the three periods (1990–2000, 2000–2010, and 2010–2018), cultivated land was mainly converted to forestland, with the change in area accounting for 21.64% of the total reduced cultivated land area. The conversion of cultivated land to forestland was predominant in the northeastern part of the KBCPW. In addition, the area of cultivated land converted to construction land during the 2010–2018 period was 124.27 km^2^, which constituted the largest proportion of construction land and was mainly distributed in the eastern part of the study area (Figure 3a). Grassland exhibited varying trends during the three periods (Figure 3b). Grassland was mainly converted to cultivated land between 1990 and 2000, which accounted for 60.53% of the total reduced grassland area in this period. Grassland was converted to forestland and cultivated land between 2000 and 2010 with areas of 275.41 km^2^ and 110.89 km^2^, respectively. Most of the grassland was converted to cultivated land and forestland between 2010 and 2018 with areas of 113.51 km^2^ and 61.33 km^2^, respectively. The area under forestland (Figure 3c) decreased slightly during the 1990–2000 period and then increased due to decreases in grassland and cultivated land, which was associated with the conversion of farmland to forestland in 2002. Moreover, marked changes were observed between 2010 and 2018 in which 537.55 km^2^ of forestland was converted to cultivated land, which led to a decline in forestland area. In addition, the areas of cultivated land and grassland converted to construction land changed significantly between 2010 and 2018.

### 3.2. Changes in Landscape Pattern Indices

**Figure 4:**
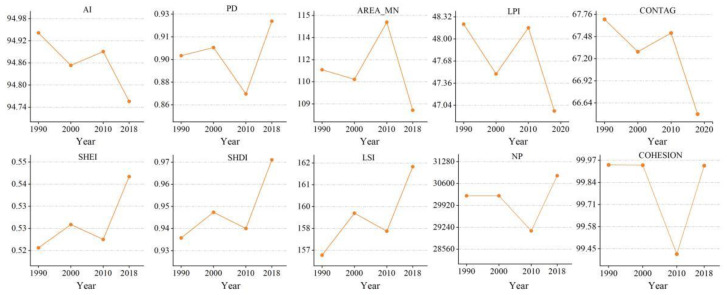
Changes in landscape metrics during the 1990–2018 period.

Landscape metric changes at the landscape level during the 1990–2018 period (Figure 4) revealed that NP and PD increased, while AREA_MN decreased, indicating that the landscape tended to fragment. LPI decreased by 2.59%, suggesting that the influence of the largest patch in the landscape gradually declined. LSI initially decreased and subsequently increased, suggesting that the landscape pattern trended to became irregular and then gradually diversified from 2000 to 2018. A decrease in aggregation and connectivity of patches was observed with CONTAG decreasing by 1.77% during 1990–2018, indicating an increase in the number of patches in the landscape and a decrease in patch aggregation and connectivity. During the study period, AI remained at a high value, indicating that with the same type of patch connection, the landscape was less fragmented. COHESION had a high value, which declined slightly in 2010, suggesting a decline in the clustering of patches. SHDI and SHEI reflected the richness of species and proportions of patch types in the landscape, with a high value indicating more patch types or the closeness of the proportions of the various landscape components. SHDI and SHEI increased by 0.0335 and 0.187, respectively, reflecting a decrease in heterogeneity of the landscape structure and a certain equilibrium trend.

**Figure 5:**
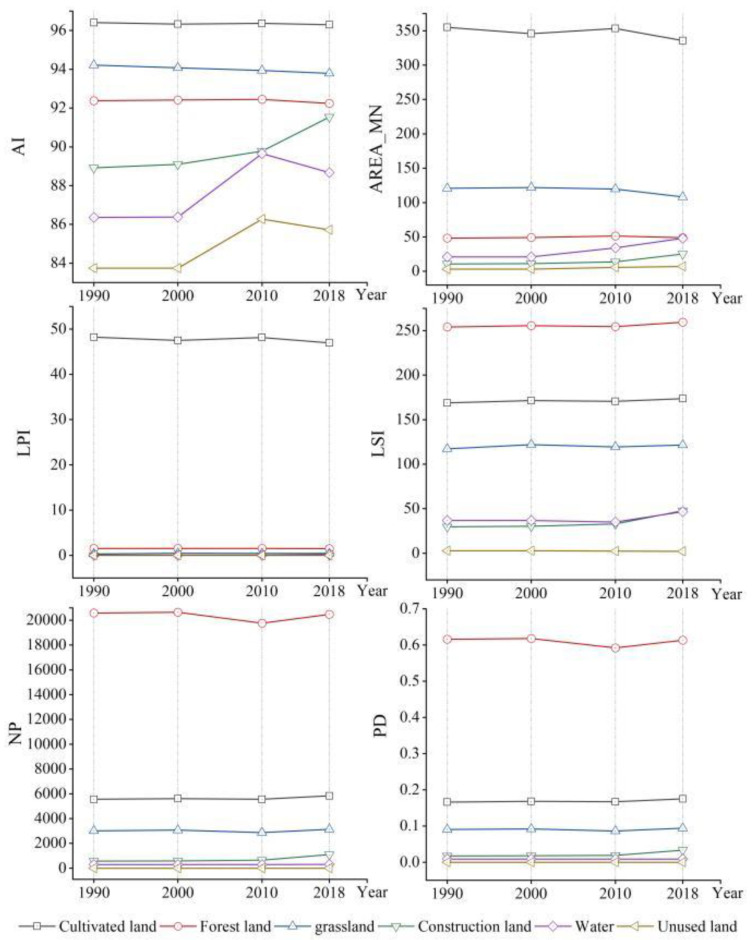
Landscape pattern indices based on class metrics (1990–2018).

At the class level (Figure 5), according to the changes in LPI, cultivated land was the dominant landscape in the KBCPW during the 1990–2018 period, followed by forestland. AREA_MN, AI, and LPI of cultivated land decreased during the study period. However, PD, LSI, and NP increased by 5.42%, 2.82%, and 4.97%, respectively. The results revealed that cultivated land had been shrinking, which was characterized by spatial segmentation, shape complexity, and decreased landscape dominance. PD, NP, and AI of forestland decreased, however AREA_MN and LSI increased by 1.32% and 2.15%, respectively, suggesting that forested areas reduced, landscape shape became more complex, and landscape aggregation reduced. The increase in NP and PD of grassland and the decrease in AREA_MN indicated that grassland fragmentation increased during the 1990–2018 period. NP, AREA_MN, and AI of water and construction land were low, indicating that water and construction land were scattered to a smaller extent.

### 3.3. Habitat Quality during the 1990–2018 Period

The HQ values generated based on the InVEST model showed continuous variation from 0 to 1. A value closer to 1 indicated higher biodiversity. For comparative analysis, the HQ of the study area was graded into four levels using the equidistant grading method [17,42] as follows: low (0–0.25), medium (0.25–0.5), relatively high (0.5–0.75), and high (0.75–1). The habitat area and percentage of each grade over the four periods were determined (Table 6).

The mean HQ values of the KBCPW during the 1990–2018 period were 0.812, 0.807, 0.809, and 0.805 at each time node, respectively. Overall, the HQ of the KBCPW exhibited a decreasing trend from 1990 to 2018. As the proportions of grass-, forest-, and cultivated lands in the region were relatively stable, the overall habitat change was negligible. Based on time, the proportion of low-level areas increased continuously during the study period, with a considerable increase being observed from 0.36% in 1990 to 0.99% in 2018. Moreover, the proportions of relatively high-level and high-level areas decreased by 0.41% and 0.46%, respectively, during the 1990–2020 period (Table 6). The high-level habitats decreased by 339.44 km^2^ between 1990 and 2000, which was associated with the decline in forestland in the study area. However, the areas of all the other three land types exhibited upward trends. The two types exhibited similar trends from 2000 to 2010 and from 2010 to 2018, suggesting that medium-level and relatively high-level areas decreased. High-level areas initially increased and then decreased, whereas low-level areas increased significantly.

**Figure 6:**
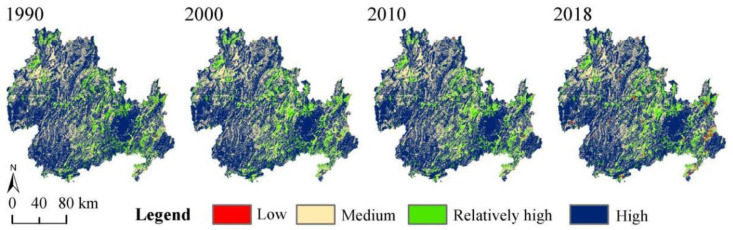
Spatial distribution and changes in habitat quality in the KBCPW during the 1990–2018 period.

On a spatial scale (Figure 6), a significant difference was observed in spatial variability during the 1990–2018 period. The high HQ value was 0.75–1, which was predominantly observed in forestland and grassland. Conversely, the low HQ value was 0–0.25, which was observed in construction land and rural settlements. A medium HQ value was mainly observed in cultivated land. Although 91.8% of the habitats remained virtually unchanged, the HQ of 4.7% of the habitats decreased during the study period. Improved HQ was mainly observed in cultivated land.

**Figure 7:**
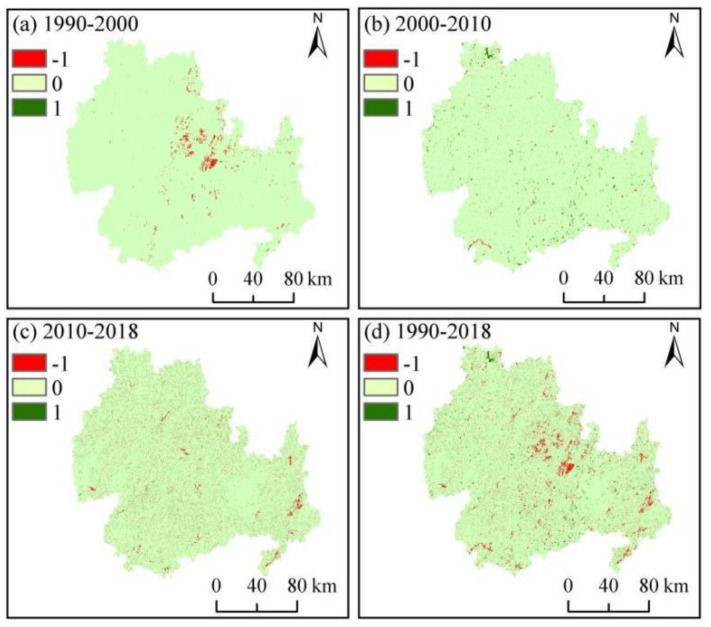
Changes in the spatial patterns of habitat quality during the 1990–2018 period.

To gain an in-depth understanding of the spatiotemporal changes in the characteristics of HQ, ArcGIS was used to obtain a map of HQ changes (Figure 7). HQ remained stable in most parts of the study area from 1990 to 2000; however, considerable habitat degradation was observed in the northeastern part of the KBCPW. HQ increased slightly in most parts of the study area from 2000 to 2010 but decreased in the southwestern part. HQ decreased significantly, particularly in the eastern region of the study area.

**Figure 8:**
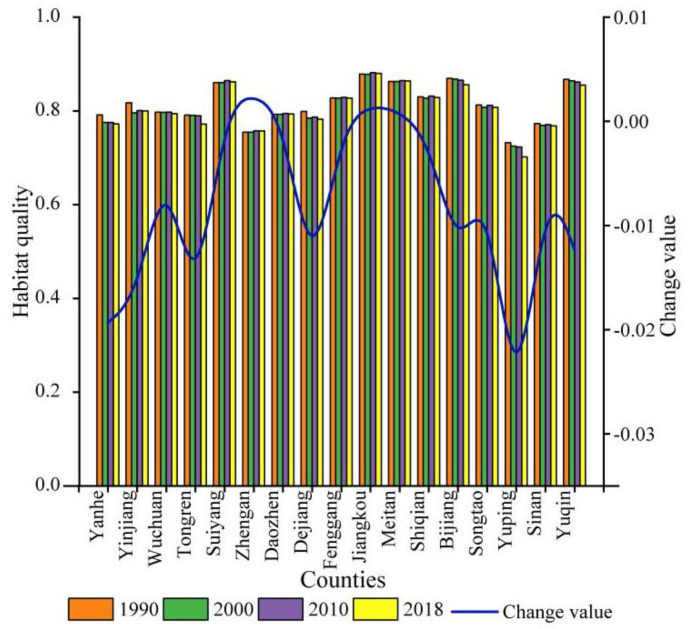
Variations in habitat quality across counties in the KBCPW.

The HQ changes observed in the KBCPW over the 1990–2018 period are illustrated in Figure 8. According to the results, HQ in Jiangkou was the highest (HQ > 0.87 annually) and the lowest was in the Yuping County (HQ of approximately 0.7 annually). During the study period, the HQ value of each county changed to varying degrees, with the most notable change being observed in the Yuping County, where the HQ value decreased by 4.11% (from 0.7322 to 0.7015). The HQ values for the Yinjiang, Tongren, Dejiang, Bijiang, and Yuqing Counties ranged from 0.012 to 0.019. The variations in HQ values for the Wuchuan, Suiyang, Zheng’an, Daozhen, Fenggang, Jiangkou, Meitan, Shiquan, Songtao, and Sinan Counties were relatively small.

### 3.4. Topographic Gradient Effect of Habitat Quality

**Figure 9:**
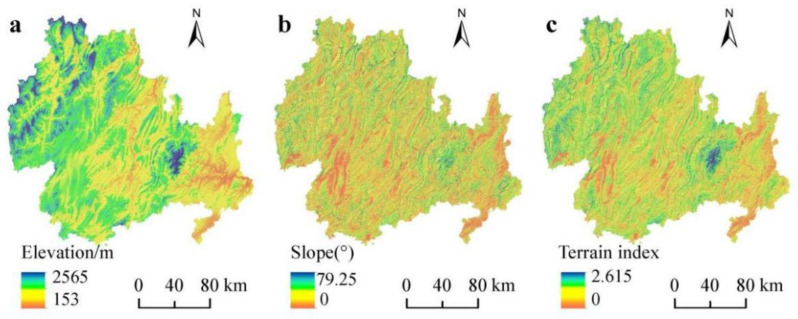
Elevation, slope, and terrain indices across various parts of the KBCPW.

**Figure 10:**
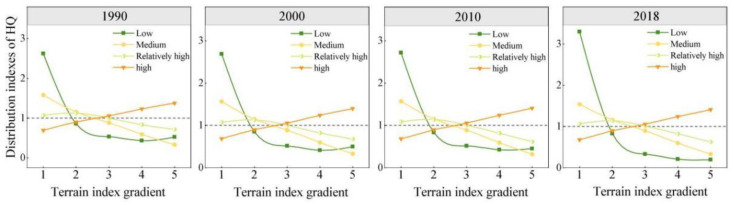
Distribution indices of habitat quality with terrain index gradient.

Topography is a key factor that affects the distribution pattern of populations and maintains community diversity [39]. The topography of the KBCPW was relatively complex, and the spatial distribution of its HQ and landscape pattern could have been influenced by topography to a great extent. Based on the elevation and slope of the study area, terrain index (Figure 9) and HQ distribution index (Figure 10) of the KBCPW were calculated according to Equations (4) and (5) to explore the distribution characteristics of HQ along a topographic gradient and to reveal the effect of topographic gradient on HQ spatial patterns.

Studies have shown that at levels 1–3 of the terrain gradient, the distribution index for low, medium, and relatively high HQ is greater than 1, which is absolutely dominant. In terrain level 3, the distribution index of relatively high and high HQ is greater than 1, which is an absolute advantage. Therefore, terrain level 3 with a mixed gradient and high HQ is an absolute advantage. The distribution of medium, relatively high, and high HQ in the study area was even across different topographic gradients during the 1990–2018 period, while that of low HQ varied slightly. The advantage of low HQ over low terrain increased, with a distribution index of 3.3 being observed. Overall, high HQ index increased, whereas low, medium, and relatively high HQ indices decreased with increasing topographic gradient at terrain level 3 and above. The topography of the study area was a key factor influencing the spatial distribution pattern of HQ, which could be explained by the fact that human activities have different spatial impacts on land use due to the variations in landform morphology [43]; this, in turn, leads to the different spatial distribution patterns of HQ. Therefore, the effect of topographical factors should be considered based on the local habitat conditions when implementing habitat protection, ecological planning, and regulation strategies.

## 4. Discussion

The ecological environment in mountainous areas is harsh and habitats are poor due to the impact of human activities and natural factors. Specifically, the contradiction between humans and land in mountainous areas dominated by karst ecosystems is prominent. Mountain ecosystems are fragile, and high habitat heterogeneity and severe rocky desertification enhance their fragility [44,45]. In this study, the KBCPW in Guizhou Province was considered an example of a karst plateau mountainous area. Our results revealed variations in the spatiotemporal characteristics of HQ in the KBCPW from 1990 to 2018, which are caused by land-use change and the distribution characteristics of HQ from a topographic perspective. The results provide a scientific basis for territorial space planning and habitat protection in mountainous areas, which is crucial for the rational and sustainable use of land resources, as well as the construction of ecological civilization.

According to the results of the present study, land-use change in the KBCPW over the 1990–2018 period was characterized by an increase in construction and cultivated lands, and a decrease in forestland and grassland. The main land-use changes in the KBCPW were the conversion of cultivated land to construction land and forestland, and the mutual conversion between grassland and forestland. These changes improved landscape heterogeneity and the degree of fragmentation. The HQ of the KBCPW was generally high, with a significant spatial difference being observed. Furthermore, the high HQ was mainly distributed in forestland and grassland, while low and medium HQ were distributed in cultivated and construction lands. With regard to the topographic gradient, the topography of the study area is complex, characterized by a large topographic gradient in the northwest and southern edges and central areas, and a relatively small spatial pattern of topographic gradient in the eastern region, which still reflects the supporting or limiting effect of geographical environment, such as topography and geomorphology, on HQ and change [46]. Low HQ was dominant in low topographic gradients, whereas high HQ was dominant in high topographic gradients, which could be because HQ is correlated with terrain and land-use type. Mountainous terrain varies greatly, and changes in elevation gradients lead to vertical distribution patterns of vegetation [47]. Therefore, the elevational distribution of regional HQ depends on the spatial distribution of LULC. In addition, the results of the present study revealed that LULC changes substantially affected HQ.

Regional HQ has a direct impact on local human welfare. With increased socioeconomic development and population growth, the demand for land and the impact of human interference on habitats are increasing. Despite the implementation of policies, such as "returning cultivated land to forestland" and "closing mountains through reforestation", the HQ in the KBCPW has improved significantly. The LULC in the study area underwent drastic changes from 1990 to 2018: the area of forestland decreased by 134.4km^2^, the shape of forestland became more complex, and the degree of agglomeration decreased; the area of grassland decreased by 241km^2^ and the fragmentation of grassland patches increased; and the construction land area increased rapidly by 4.5-fold and cultivated land increased by 0.23%. Rapid urbanization and a large amount of cultivated land demand have made a large number of forestland and grassland areas in this area occupied by construction land and cultivated land, resulting in habitat loss and fragmentation, which, in turn, has led to an increase in low HQ areas in the region. The KBCPW experienced habitat degradation during the 1990–2018 period. The areas with HQ decrease are largely located in the northeastern and eastern regions of the KBCPW due to the accelerated urbanization, rapid expansion of construction land, and the loss of large areas of forestland. The changes in land use consequently threaten the surrounding habitats, leading to increased habitat fragmentation and poor connectivity, and ultimately habitat degradation in the region. The areas with improved HQ are located in the southern and northern parts of the KBCPW, which could be due to the implementation of projects, such as “returning cultivated land to forestland” and “closing mountains for afforestation and grass cultivation”. Although conservation policies promote the improvement of ecological environments in some areas, the positive impact of policy interventions on environmental protection cannot compensate for the negative impact of human activities. Therefore, to promote a balance between sustainable development and environmental protection, political and economic policies should be formulated from the perspective of ecosystems.

The InVEST model is relatively mature and has certain advantages over other traditional methods in terms of spatial expression and dynamic research [39,48]; however, there is a certain subjectivity in the parameter setting in the calculation, and the parameter verification and rationality evaluation are worth discussing in depth. The HQ assessment results obtained using the InVEST-HQ model depended on land-use classification. Land use was classified into six categories and the datasets did not take into account the internal heterogeneity of each land-use type. In the present study, the model is based on the assumption that regions with high HQ have high biodiversity, while regions with low HQ have low biodiversity [35]. However, in reality, areas with good regional HQ do not necessarily have a rich species diversity and the principles of the module are more inclined toward vegetation diversity, which presents certain limitations when calculating regional HQ [13]. Although the InVEST model has limitations in assessing HQ, it can be calculated by inputting land-use parameters, thereby providing basic information for guiding ecological environment protection.

## 5. Conclusions

Understanding the spatial and temporal changes of HQ is of great significance for regional sustainable development. The present study used the InVEST model to explore the spatiotemporal variations in HQ and its response to topographic gradient effect in the KBCPW in Guizhou Province during the 1990–2018 period. The results were as follows:

(1) Cultivated land, grassland, and forestland were the main land-use type in the KBCPW during the 1990–2018 period. The major land-use changes in the study area were the conversion of cultivated land to construction land and forestland and the conversion of forestland to cultivated land and grassland.

(2) Cultivated land was the dominant landscape in the KBCPW, followed by forestland. Landscape patches tended to fragment, which, in turn, shaped complexity and variety and decreased connectivity. Land-use types exhibited varying trends in their landscape patterns.

(3) The HQ of KBCPW showed a downward trend from 1990 to 2018, although the overall HQ was high, which was attributed to the high HQ of the areas covered. Spatial differentiation in the study area was significant. High HQ was observed in areas dominated by forestland, while relatively low HQ was observed in construction and cultivated lands, which were the major land-use types and were closely associated with human activities.

(4) HQ exhibited varying spatiotemporal patterns with topographic gradients and HQ degradation was predominantly distributed in low terrain areas. Generally, HQ degradation in low terrain areas was relatively high, slight degradation was observed in the medium terrain areas, and HQ in the high terrain areas was stable.

## Figures and Tables

**Table 1:** Land-use classification system.

Primary Land-Use Type	Secondary Land-Use Type
Cultivated land	Paddy land, Dry land
Forest land	Forest, Shrub, Open Forest, Other forests
Grassland	High-coverage grassland, Moderate-coverage grassland, Low-coverage grassland
Water	River canalization, Lake, Reservoir and Pond, Beach land
Construction land	Urban land, Rural settlement, Other Construction land
Unused land	Bare land

**Table 2:** Threat factors and maximum distance of influence, weights of threat factors, and decay types identified in the study area.

Threat Factors	Max Distance of Influence/km	Weights	Decay Type
Cultivated land	5	0.7	Linear
Urban land use	8	1.0	Exponential
Rural settlements	5	0.6	Exponential
Other construction land	3	0.5	Exponential

**Table 3:** Habitat suitability and sensitivity of land-use type to each threat factor.

Land Code	Land-Use Type	Habitat Suitability	Cultivated Land	Urban Land Use	Rural Settlements	Other Construction Land
11	CL	0.5	0	0.7	0.6	0.5
12	FL	1	0.8	0.85	0.9	0.6
21	SL	1	0.5	0.6	0.65	0.5
22	OFL	1	0.9	0.8	0.9	0.7
23	OWL	1	0.9	0.85	0.85	0.7
24	HCG	0.8	0.6	0.6	0.55	0.2
31	MCG	0.7	0.55	0.7	0.5	0.3
32	LCG	0.6	0.5	0.6	0.5	0.4
33	RC	0.8	0.6	0.6	0.5	0.3
41	LK	0.9	0.65	0.75	0.65	0.4
42	RP	0.7	0.6	0.6	0.5	0.5
43	BL	0.6	0.6	0.7	0.65	0.5
51	UL	0	0	0	0	0
52	RS	0	0	0	0	0
53	OCL	0	0	0	0	0
66	BL	0	0	0	0	0

Notes: CL: cultivated land; PL: paddy land; DL: dry land; FL: forestland; SL: shrubland; OFL: open forestland; OWL: other wooded land; HCG: high-coverage grassland; MCG: medium-coverage grassland; LCG: low-coverage grassland; RC: river canalization; LK: lake; RP: reservoir and pond; BL: beach land; UL: urban land; RS: rural settlement; OCL: other construction land; BL: bare land.

**Table 4:** Variations in the areas of different types of land from 1990 to 2018.

Year	Cultivated Land		Grassland		Forestland		Water		Construction Land		Unused Land	
	km^2^	%	km^2^	%	km^2^	%	km^2^	%	km^2^	%	km^2^	%
1990	9928.0	29.71	3638.8	10.89	19,723.0	59.02	62.4	0.19	60.8	0.18	0.12	0.00
2000	10,148.9	30.37	3751.9	11.23	19,383.6	58.01	62.7	0.19	65.9	0.20	0.12	0.00
2010	10,134.4	30.33	3433.3	10.30	19,659.9	58.84	97.8	0.29	88.0	0.26	0.11	0.00
2018	10,005.0	29.94	3397.8	10.17	19,588.6	58.63	147.1	0.44	274.8	0.82	0.07	0.00

**Table 5:** Land-use change matrices from 1990 to 2018 (km^2^).

Year	Land-Use Type	Grassland	Cultivated Land	Construction Land	Forestland	Water	Unused Land
1990–2000	grassland	3560.53	68.47	0.98	8.77	0.00	0.00
	Cultivated land	0.49	9919.82	4.13	3.20	0.32	0.00
	Construction land	0.00	0.00	60.78	0.00	0.00	0.00
	Forestland	190.82	160.54	0.03	19,371.56	0.00	0.00
	Water	0.00	0.00	0.00	0.01	62.36	0.00
	Unused land	0.00	0.00	0.00	0.00	0.00	0.12
2000–2010	grassland	3360.78	110.89	2.97	275.41	1.47	0.00
	Cultivated land	50.36	9851.19	11.97	223.88	11.16	0.00
	Construction land	0.13	1.04	64.30	0.31	0.13	0.00
	Forestland	21.54	170.18	8.67	19,157.54	24.00	0.00
	Water	0.19	0.63	0.05	0.78	60.96	0.00
	Unused land	0.00	0.00	0.00	0.00	0.00	0.11
2010–2018	grassland	3216.96	113.51	34.92	61.33	5.23	0.00
	Cultivated land	110.89	9345.51	124.27	532.47	19.53	0.00
	Construction land	0.99	4.30	80.21	1.84	0.61	0.00
	Forestland	66.92	537.55	34.82	18,980.53	31.95	0.00
	Water	0.65	2.51	0.55	4.50	89.24	0.00
	Unused land	0.00	0.00	0.00	0.00	0.04	0.05

**Table 6:** Proportions of each habitat level from 1990 to 2018.

HQ Grades	Value range	1990	2000	2010	2018	Change/%
		Proportion/%	Proportion/%	Proportion/%	Proportion/%	
Low	0–0.25	0.36%	0.37%	0.44%	0.99%	0.63
Medium	0.25–0.5	29.66%	30.32%	30.28%	29.89%	0.23
Relatively high	0.5–0.75	9.92%	10.26%	9.56%	9.51%	−0.4
High	0.75–1	60.06%	59.05%	59.73%	59.60%	−0.46
Mean HQ		0.812	0.807	0.809	0.805	−0.007

## Data Availability

Not applicable.
